# UAV Path Planning Algorithm Based on Improved Harris Hawks Optimization

**DOI:** 10.3390/s22145232

**Published:** 2022-07-13

**Authors:** Ran Zhang, Sen Li, Yuanming Ding, Xutong Qin, Qingyu Xia

**Affiliations:** 1 School of Information Engineering, Dalian University, Dalian 116622, China; nancy444@163.com (R.Z.); q1533664997@163.com (X.Q.); xiaqingyu0315@163.com (Q.X.); 2 Communication and Network Laboratory, Dalian University, Dalian 116622, China; dingyuanming@dlu.edu.cn

**Keywords:** flight path planning, Harris Hawks optimization, Cauchy mutation strategy, adaptive weight, sine-cosine algorithm, unmanned aerial vehicle system

## Abstract

In the Unmanned Aerial Vehicle (UAV) system, finding a flight planning path with low cost and fast search speed is an important problem. However, in the complex three-dimensional (3D) flight environment, the planning effect of many algorithms is not ideal. In order to improve its performance, this paper proposes a UAV path planning algorithm based on improved Harris Hawks Optimization (HHO). A 3D mission space model and a flight path cost function are first established to transform the path planning problem into a multidimensional function optimization problem. HHO is then improved for path planning, where the Cauchy mutation strategy and adaptive weight are introduced in the exploration process in order to increase the population diversity, expand the search space and improve the search ability. In addition, in order to reduce the possibility of falling into local extremum, the Sine-cosine Algorithm (SCA) is used and its oscillation characteristics are considered to gradually converge to the optimal solution. The simulation results show that the proposed algorithm has high optimization accuracy, convergence speed and robustness, and it can generate a more optimized path planning result for UAVs.

## 1. Introduction

With the rapid development of the communication technology, sensors, artificial intelligence and 5G technology, the Unmanned Aerial Vehicle (UAV) plays a crucial role in modern military war [1]. UAV path planning is a key problem in UAV systems [2], and the quality of UAV path directly determines the success or failure of combat missions. Therefore, it is of great significance to study UAV path planning algorithms in complex combat environment.

Many experts and researchers have performed in-depth studies on UAV path planning. According to the dimension of the planning space, it is mainly divided into two-dimensional (2D) [3] and three-dimensional (3D) path planning [4]. The developed model for 3D path planning is stereoscopic, and considers topography and threat factors, which is closer to the actual environment. However, it increases the complexity of path planning. The UAV 3D path planning algorithms mainly include classical algorithms and swarm intelligence algorithms [5]. The classical algorithms include the A-Star algorithm [6], Differential Evolution (DE) [7], Dijkstra algorithm [8] and simulated annealing [9]. Although these algorithms have their own advantages, they all have some disadvantages, such as the long search time and large memory consumption. The swarm intelligence algorithm [10] forms a self-organizing and adaptive stochastic optimization algorithm with bionic behavior by observing the living habits, foraging behaviors and social characteristics of the biological populations. Common swarm intelligence algorithms for path planning include the Particle Swarm Optimization (PSO) [11], Firefly Algorithm (FA) [12], Ant Colony optimization (ACO) [13], Artificial Bee Colony (ABC) algorithm [14] and Whale Optimization Algorithm (WOA) [15]. The swarm intelligence algorithm has become the most widely studied approach in the UAV path planning field, due to its better robustness, flexibility and high search accuracy.

Ji et al. [16] propose a new double-dynamic biogeography-based learning particle swarm optimization that use the double-dynamic biogeography-based learning strategy replacing the traditional learning mechanism from the personal and global best particles to select the learning particles in order to learn from better objects and maintain the ability of jumping out of local optimality. He et al. [17] propose a novel hybrid algorithm called HIPSO-MSOS by combining improved particle swarm optimization (IPSO) and modified symbiotic organisms search (MSOS) and adopt the time stamp segmentation (TSS) model to simplify the handling of coordination cost of UAVs. The exploration and exploitation abilities are improved efficiently, which brings good performance to the proposed algorithm. Xia et al. [18] propose a novel multi-objective PSO algorithm (GMOPSO-QL) that adopt the Gaussian distribution-based updating operator to generate new particles, introduce the exploration and exploitation modes to enhance population diversity and convergence speed, respectively and introduce the Q-Learning based mode selection logic to balance the global search with the local search in the evolution process. Yu et al. [19] propose a sparrow PSO algorithm that selects a suitable model for path initialization, changes the discoverer position update and reinforces the influence of start-end line on path search, which can significantly reduce blind search and increase the number of target points reached by adaptive variable speed escapes in areas of deadlock. Liu et al. [20] propose an optimal mission assignment and path planning method of multi-UAV for disaster rescue which build three threat sources and a cost-revenue function, then design an adaptive genetic algorithm (AGA) to solve the mission allocation task and propose a fitness function which considers the current and maximum iteration numbers to improve the AGA convergence performance. Zhang et al. [21] propose an improved adaptive grey wolf optimization algorithm that propose an adaptive convergence factor adjustment strategy and an adaptive weight factor to update the individual’s position, establish the environmental map model by integrating digital elevation map and equivalent mountain threat model, and the performance evaluation function is established by fitting the calculated track length. Liu et al. [22] propose a fusion of Sparrow Search Algorithm (SSA) and Bioinspired Neural Network that use SSA to find a series of nodes with the lowest comprehensive cost on the safe surface and when the dynamic obstacle is detected in the predetermined trajectory, the improved BINN method is used to carry out local path re-planning to achieve the purpose of dynamic obstacle avoidance. Tong et al. [23] propose an improved method of path planning and autonomous formation for UAVs that devise the mathematical model for UAV path planning as a multi-objective optimization with three indices and develop the method integrated by pigeon-inspired optimization and mutation strategies of differential evolution to optimize feasible paths. Huo et al. [24] propose a hybrid differential symbiotic organisms search (HDSOS) algorithm that the concept of traction function is put forward and used to improve the efficiency and a perturbation strategy is adopted to further enhance the robustness of the algorithm. Based on the characteristics of the standard BA and the artificial bee colony algorithm (ABC), Zhou et al. [25] propose a new modification of the BA algorithm that the improved bat algorithm integrated into the ABC algorithm and use ABC to modify the BA and solve the problem of poor local search ability of the BA. However, these swarm intelligence algorithms still have some defects, such as slow convergence speed, falling easily into local optimal solution, high dependence on excellent individuals and complex parameter settings. 

Harris Hawks Optimization (HHO) is a new swarm intelligence algorithm proposed by Heidari et al. [26]. This algorithm is inspired by Harris Hawks’ predation behavior, and includes two stages of search and development. Compared with other swarm intelligence algorithms, HHO has simpler principle, fewer parameters and stronger global exploration ability. However, similar to other swarm intelligence algorithms, HHO has slow convergence speed, low optimization accuracy and it can easily fall into local optimization when solving complex optimization problems. Therefore, Guo et al. [27] improve HHO using the good point set and nonlinear convergence equation. Zhang et al. [28] introduce the exponential decline strategy to update the energy factor. Kamboj et al. [29] propose the hybrid sine-cosine and HHO to increase the global exploration ability of the algorithm. Fan et al. [30] combine HHO and the quasi-reflection-based learning mechanism (QRBL) together in order to improve the convergence speed and solution accuracy. Zou et al. [31] propose an adaptive relative reflection HHO (ARHHO), which increases the diversity of the standard HHO, alleviates the problem of stagnation of local optimal solutions, and improves the search accuracy of the algorithm. Hussien et al. [32] enhance the performance of HHO by combining HHO with opposition-based learning (OBL), Chaotic Local Search (CLS) and a self-adaptive technique. 

In order to further improve the quality and efficiency of UAV path planning, this study proposes a path planning algorithm based on Sine-cosine and Cauchy combined HHO (SCHHO). The Cauchy mutation strategy [33] is used to improve the global exploration ability, and the adaptive weight [34] is introduced to improve the exploitation capacity of the proposed algorithm. In order to reduce the possibility of falling into local extremum, the Sine–cosine Algorithm (SCA) [35] is added, and its oscillation characteristics are used to gradually converge to the optimal solution. In addition, this study develops a complex real terrain model, uses terrain and threat information to cut the path planning space, and introduces the maximum range constraint to reduce the search range, so as to achieve fast and accurate planning of feasible paths. Since the fixed wing generally needs a runway or catapult to launch, has no vertical take-off capability, and this UAV system cannot hover, the UAV in this paper is assumed to be a multi-rotor UAV, and its speed and altitude are adjusted according to the terrain and nodes.

The remainder of this paper is organized as follows. Section 2 presents the modeling and constraints of path planning. Section 3 details the proposed UAV path planning algorithm based on improved HHO. Section 4 shows the experimental results and analysis. Finally, the conclusion and future work are drawn in Section 5.

## 2. Modeling and Constraints

### 2.1. Environmental Modeling

In order to study the UAV path planning problem, it is necessary to develop a model similar to the real combat environment, that is, a 3D digital map containing reference terrain, obstacle and threat area information. The terrain is simulated by numerical coding, and the peaks and valleys are presented in a matrix form. More precisely, the matrix values represent the terrain elevation under the current coordinate position. Finally, the terrain is smoothly simulated using the interpolation method.

**Figure 1:**
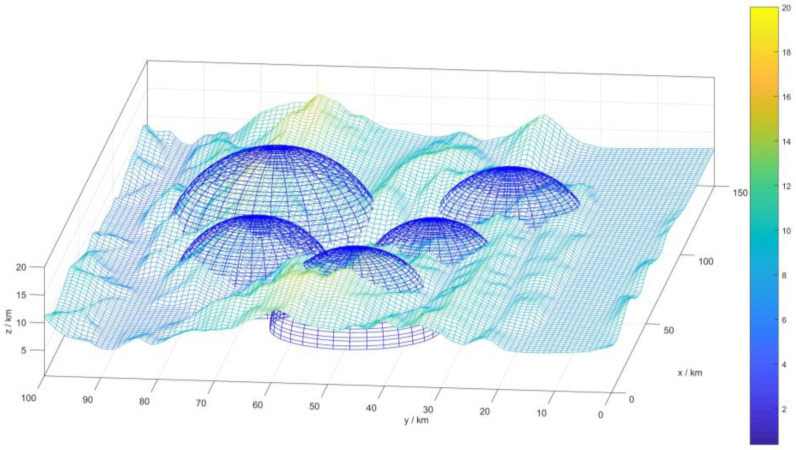
3D digital map.

In the UAV combat environment, many dangerous zones, that are referred to as threat areas, exist. This study considers the enemy’s radar detection [20] and takes its detection range as the threat area. The calculation of the radar detection area is expressed in Equation (1), and the blue hemispherical model (cf. Figure 1) is used to model these threat areas.

(1) $$W_{i}(x,y,z)=\{\begin{array}{c} \sum_{i}{\left(x-x_{i}\right)}^{2}+{\left(y-y_{i}\right)}^{2}+{\left(z-z_{i}\right)}^{2}=R_{i}^{2}\\ z\geq 0 \end{array}$$

where *W_i_*(*x*,*y*,*z*) represents the detection area of the *i*-th radar, (*x_i_*, *y_i_*, *z_i_*) is the location of the radar, and *R_i_* denotes the detection radius of the radar.

Figure 1 is a simulated modeling of the drone’s flight environment, where the blue hemispheric model represents the modeling of the threat area.

### 2.2. Path Cost Function

In order to measure the quality of the UAV planned path, it is necessary to establish a path cost function. The path cost function used in this study is given by:

(2) $$F=\sum_{i=1}^{n}\left(\omega_{1}l_{i}+\omega_{2}h_{i}+\omega_{3}f_{i}\right)$$

where *n* is the number of flight path segments, *l_i_* (*i* = 1, 2,…, *n*) is the path length of segment *i*, *h_i_* is the average flight height of segment *i*, *f_i_* is the comprehensive threat index of segment *i*, *ω*_1_, *ω*_2_ and *ω*_3_ are the corresponding weight coefficients of path length, average flight height and comprehensive threat index, respectively.

The comprehensive threat index *f_i_* can be calculated as:

(3) $$f_{i}=\frac{\sum_{j=1}^{m}Q_{ij}}{{\left(D_{ij}\right)}^{4}}$$

where *m* is the number of threat points, *Q_ij_* (*j* = 1, 2,…, *m*) represents the threat index of segment *i* relative to threat point *j* which can be collected by the control center, and *D_ij_* represents the distance between the UAV and the threat point *j* in segment *i*.

In the calculation of individual fitness, it is necessary to first normalize the values of each part in the path cost function to avoid the calculation error caused by the order of magnitude difference of each value.

### 2.3. Path Constraints

Considering the performance limitations and practical factors, the path planning of the UAV should meet certain constraints [36].

- Constraint on Minimum Path

The minimum path is defined as the minimum distance that the UAV should keep steady forward flight before changing the flight attitude. The frequent attitude changes during flight affect the stability of the UAV, and even lead to crash. Therefore, frequent attitude changes should be avoided as much as possible. The constraint is then given by:

(4) $$l_{i}>l_{\min}$$

where *l_min_* is the length of the shortest path.

- Constraint on Maximum Path

The maximum path is defined as a preset maximum length that the total flight path length should be less than or equal to, due to the fuel restriction or special mission requirements. The constraint is then given by:

(5) $$\sum_{i=1}^{n}\left|l_{i}\right|\leq L_{\max}$$

where *L_max_* is the maximum path length

- Constraint on Minimum Ground Clearance

The minimum ground clearance is defined as the minimum flight height that the UAV should meet during flight to avoid collision with the ground. The constraint is then given by:

(6) $$h_{i}\geq h_{\min}$$

where *h*_min_ is the minimum ground clearance.

- Constraint on Maximum Turning Angle

The maximum turning angle is defined as the maximum range of continuous course change of the UAV making circular motion in horizontal plane. In a complex environment, when making large angle turns, the UAV is very vulnerable to wind and other factors. Therefore, it is necessary to limit its continuous turning angle. The constraint is then given by:

(7) $$\varphi_{i}<\Delta \varphi_{\max}$$

where $\Delta \varphi_{\max}$ is the maximum turning angle and $\varphi_{i}$ (*i* = 1, 2, …, *n* − 1) is the *i*-th turn angle of the UAV.

- Constraint on Maximum Climb Angle

The maximum climb angle is defined as the angle of climb and descent that the UAV requires to be limited during flight. The constraint is then given by:

(8) $$\frac{\left|z_{i}-z_{i-1}\right|}{a_{i}}\leq \tan\theta_{\max}$$

where $\theta_{\max}$ is the maximum angle of climb for the UAV, $\left|z_{i}-z_{i-1}\right|$ is the height difference of path segment *i*, and *a*_i_ is the horizontal projection length of path segment *i*.

## 3. UAV Path Planning Algorithm Based on SCHHO

### 3.1. Overview of Basic HHO

HHO uses a mathematical formula to simulate the strategy of Harris Hawks catching prey under different mechanisms according to the real situation. In HHO, the Harris Hawk is the candidate solution, and the prey approaches the optimal solution by iteration. The HHO algorithm includes two phases: global exploration and local exploitation. HHO realizes the transition from global exploration to local exploitation through the energy equation of prey. The corresponding mathematical expression is as given by:

(9) $$E=2E_{0}\left(1-\frac{t}{T}\right)$$

(10) $$E_{0}=2*rand-1$$

where *E* represents the escape energy of prey, *E*_0_ denotes the initial state of prey energy, *T* is the maximum number of iterations, and *rand* is a random number within the range of (0,1). 

Note that, when |*E|* ≥ 1, HHO enters the global exploration phase, while when |*E|* < 1, it enters the local exploitation phase.

#### 3.1.1. Global Exploration

During the global exploration phase, Harris Hawks inspect and monitor the search space [*lb*,*ub*], and randomly search for prey randomly according to two strategies. The position is updated with probability *q* during iteration:

(11) $$X_{t+1}=\{\begin{array}{c} X_{rand}-r_{1}\left|X_{rand}-2r_{2}X_{t}\right|,q\geq 0.5\\ \left(X_{prey,t}-X_{average,t}\right)-r_{3}\left(lb+r_{4}(ub-lb)\right),q<0.5 \end{array}$$

where *X_t_*_+1_ and *X_t_* are, respectively, the positions of Harris Hawks in the (*t +* 1)-th and *t*-th iterations; *X_prey,t_* represents the positions of prey in the *t*-th iteration; *r*_1_, *r*_2_, *r*_3_, *r*_4_ and *q* are random numbers between 0 and 1; *lb* and *ub* are, respectively, the lower and upper bounds of the search space; *X_rand,t_* represents the random position of Harris Hawks in the *t*-th iteration; and *X_avergae,t_* denotes the average position of Harris Hawks with population *N* in the *t*-th iteration:

(12) $$X_{average,t}=\frac{1}{N}\sum_{i=1}^{N}X_{i,t}$$

#### 3.1.2. Local Exploitation

During the local exploitation phase, Parameter *E* is used to select the besiege strategy of the Harris Hawks. When |*E|* ≥ 0.5, soft besiege is executed, while when |*E|* < 0.5, hard besiege is performed. The probability of prey escaping is expressed by the random parameter *u* generated during initialization. When *u* ≥ 0.5, the prey escapes successfully. According to the chase strategy of Harris Hawks and the escape behavior of prey, HHO includes four strategies to simulate the chase attack behavior.

A. Soft besiege

When |*E|* ≥ 0.5 and *u* ≥ 0.5, the escape energy *E* of prey is sufficient. At this point, Harris Hawks choose to gradually consume the prey’s energy and then make a surprise dive in the best position to arrest the prey. The position update strategy is given by:

(13) $$X_{t+1}=\Delta X_{t}-E\left|JX_{prey,t}-X_{t}\right|$$

(14) $$\Delta X_{t}=X_{prey,t}-X_{t}$$

(15) $$J=2\left(1-r_{5}\right)$$

where, Δ*X_t_* is the difference between the position of Harris Hawks and prey during iteration, *J* is the random jump of prey when escaping, and *r*_5_ is a random number ranging between 0 and 1.

B. Hard besiege

When |*E|* < 0.5 and *u* ≥ 0.5, the prey is exhausted and the escape energy *E* is very low. At this time, Harris Hawks will quickly raid the prey, and the strategy of position update is expressed as:

(16) $$X_{t+1}=X_{prey,t}-E\left|\Delta X_{t}\right|$$

C. Soft besiege with progressive rapid dives

When |*E|* ≥ 0.5 and *u* < 0.5, the escape energy *E* of prey is sufficient, and Harris Hawks will establish a soft besiege before striking. Levy function (LF) [37] is integrated into HHO to simulate the jumping action and escape mode of prey. The strategy of updating position is expressed as:

(17) $$X_{t+1}=\{\begin{array}{c} Y:X_{prey,t}-E\left|JX_{prey,t}-X_{t}\right|,\mathrm{if}F(Y)<F(X_{t})\\ Z:Y+S\times LF(D),\mathrm{if}F(Z)<F(X_{t}) \end{array}$$

(18) $$LF(x)=0.01\times \frac{u\times \sigma }{{\left|v\right|}^{\frac{1}{\beta }}}$$

(19) $$\sigma ={\left(\frac{\Gamma \left(1+\beta \right)\times \sin(\frac{\pi \beta }{2})}{\Gamma (\frac{1+\beta }{2})\times \beta \times 2^{\left(\frac{\beta -1}{2}\right)}}\right)}^{\frac{1}{\beta }}$$

where *D* is the dimension of the problem and *S* is a random vector of size 1 × *D*, *u* and *v* are random values ranging between 0 and 1, and *β* is a default constant set to 1.5.

D. Hard besiege with progressive rapid dives

When |*E|* < 0.5 and *u* < 0.5, the prey’s escape energy *E* is insufficient, and Harris Hawks capture the prey by constructing a hard besiege before striking, so as to reduce the average position distance between it and the escaping prey. The position update strategy is given by:

(20) $$X_{t+1}=\{\begin{array}{c} Y:X_{prey,t}-E\left|JX_{prey,t}-X_{m,t}\right|,ifF(Y)<F(X_{t})\\ Z:Y+S\times LF(D),ifF(Z)<F(X_{t}) \end{array}$$

In summary, HHO uses energy *E* and factor *u* to regulate the four kinds of hunting mechanisms between Harris Hawks and prey, so as to perform the optimal solution of the problem.

### 3.2. Improved Sine-Cosine and Cauchy Combined HHO

Since the traditional HHO has some defects in its structure, the search process is prone to fall into local optimum and it has a low convergence accuracy. Therefore, the Cauchy mutation strategy, adaptive weight and SCA function are added, so as to increase the diversity of Harris Hawk population, improve the search speed and enhance the search ability of the HHO algorithm. 

#### 3.2.1. Cauchy Mutation Strategy

In the global exploration stage, the Cauchy distribution function is used to increase the diversity of Harris Hawk population, increase the search space and improve the global exploration ability of the algorithm. Combined with Cauchy operator, the mutation effect at both ends of the Cauchy distribution function is fully used in order to optimize the global optimal object. The standard the Cauchy distribution function is expressed as:

(21) $$f\left(x\right)=\frac{1}{\pi }\left(\frac{1}{x^{2}+1}\right)$$

Since the peak value of Cauchy function is relatively small, Harris Hawks will search a more global optimal value after Cauchy mutation, and use less time to explore local interval. In addition, since the Cauchy function gently declines from the peak to the sides, after updating the position by Cauchy variation, the Harris Hawk becomes less constrained by the local extreme point, and it can jump out of the local extreme point. Using the mathematical model of Cauchy variation, the current global optimal solution *X_best_* is updated as:

(22) $$X\prime_{best}=X_{best}+X_{best}\times Cauchy(0,1)$$

#### 3.2.2. Adaptive Weight

In the local exploitation stage, an adaptive weight method is introduced to update the neighborhood of prey location, so as to improve the local exploitation ability. In this study, the adaptive weight set belongs to an inertial weighting factor. When the inertial weighting factor is large, the algorithm spends more time on global exploration. When the inertia weighting factor is small, the local exploitation time is relatively long, and the optimal solution can be better determined.

The adaptive weight *ω* and prey position update are expressed as:

(23) $$\omega =\sin\left(\frac{\pi \times t}{2T}+\pi \right)+1$$

(24) $$X\prime_{prey}=\omega \times X_{prey}$$

where *T* is the maximum number of iterations, and *t* is the current number of iterations. 

In the four besiege mechanisms, the position of prey is updated with smaller adaptive weight, in order to improve the local optimization ability of the proposed algorithm.

#### 3.2.3. Sine-Cosine Algorithm

In the process of Harris Hawk predation, the location of prey plays a crucial role, which affects the forward direction of the entire Harris Hawk population. However, when the prey searched by Harris Hawks is located in the local optimal position, a large number of followers will flock to this position. At this time, the discoverer and the whole population will stagnate, which results in the loss of population location diversity, and then the possibility of falling into the local extreme value is increased. To solve this problem, this study introduces the SCA in the location update of HHO. SCA consists in using the oscillation characteristics of sine and cosine function to gradually converge to the optimal solution, so as to obtain the overall optimal value.

SCA divides the optimization process into two stages: exploration and exploitation. The global optimal solution is approached continuously approached through these two stages. The position update equations of the two stages of SCA are given by:

(25) $$x_{i,j}^{t+1}=x_{i,j}^{t}+r_{6}\times \sin(r_{7})\times \left|r_{8}\cdot x_{best}-x_{i,j}^{t}\right|,r_{9}<0.5$$

(26) $$x_{i,j}^{t+1}=x_{i,j}^{t}+r_{6}\times \cos(r_{7})\times \left|r_{8}\cdot x_{best}-x_{i,j}^{t}\right|,r_{9}\geq 0.5$$

where, *x_best_* is the global extremum on the *i*-dimension of the *t*-th iteration, $x_{i,j}^{t}$ is the position of the *j*-th solution on the *i*-dimension of the *t*-th iteration, *r*_7_ is a random number in the range [0, 2π], *r*_8_ is a random number in the range [−2, 2], *r*_9_ is a random number in the range [0, 1] and *r*_6_ is a linearly decreasing function expressed as:

(27) $$r_{6}=a-t\frac{a}{T}$$

where, *t* is the current iteration number, *T* is the maximum number of iterations and *a* is a constant, usually equal to 2.

According to Equations (25) and (26), SCA mainly includes four parameters: *r*_6_, *r*_7_, *r*_8_, and *r*_9_. *r*_6_ determines the direction in which Harris Hawks will move next and controls the transformation from exploration stage to exploitation stage. *r*_7_ determines how far Harris Hawks travel. *r*_8_ enhances or weakens the influence of the move direction. *r*_9_ makes Equations (25) and (26) randomly switch when the position is updated.

Based on these procedures and analyses, the pseudo-code of SCHHO is presented in Algorithm 1.
**Algorithm 1** SCHHO**Inputs**: Population size *N* and maximum number of iterations *T***Outputs**: Location of prey and its value of fitnessInitialize the random population *X_i_* (*i* = 1; 2; …; *N*)**While** (*t* < *T*)     Calculate the fitness value of Harris hawks;     Set the parameter *X_prey_* as the best position of the prey;     **for** (each Harris hawks *(X_i_*)) **do**     Update the initial energy *E*_0_ and jump strength *J* using Equations (10) and (15);     Update *E* using Equation (9);     **if** (|*E*| ≥ 1) **then**          // Exploration phase     Update the location vector using Equations (11) and (22);     **if** (|*E*| < 1) **then**          // Exploitation phase     **if** (*u* ≥ 0.5 and |*E*| ≥ 0.5) **then**   // Soft besiege     Update the location vector using Equation (13);     **if** (*u* ≥ 0.5 and |*E*| < 0.5) **then**   // Hard besiege     Update the location vector using Equation (16);          **if** (*u* < 0.5 and |*E*| ≥ 0.5) **then**   // Soft besiege with progressive rapid dives     Update the location vector using Equation (17);          **if** (*u* < 0.5 and |*E*| < 0.5) **then**   // Hard besiege with progressive rapid dives     Update the location vector using Equation (20);     **end**     Update the location vector using Equation (24);     **end**    **end** **end**Initialize the starting position of the search agents using the final position obtained by the Harris Hawks optimizer;**Do**     Evaluate each of the search agents using objective functions;     Update the best fitness obtained so far;     Update the random numbers *r*_6_, *r*_7_, *r*_8_ and *r*_9_; **if** (*r*_9_ < 0.5)     Update the position of search agents using Equation (25);     **else**     Update the position of search agents using Equation (26); **end** **While** (*t* < *T*) **Return** the best optimal solution;     Record the mean, best optimal solution and standard deviation.

### 3.3. Path Planning Based on Improved SCHHO

**Figure 2:**
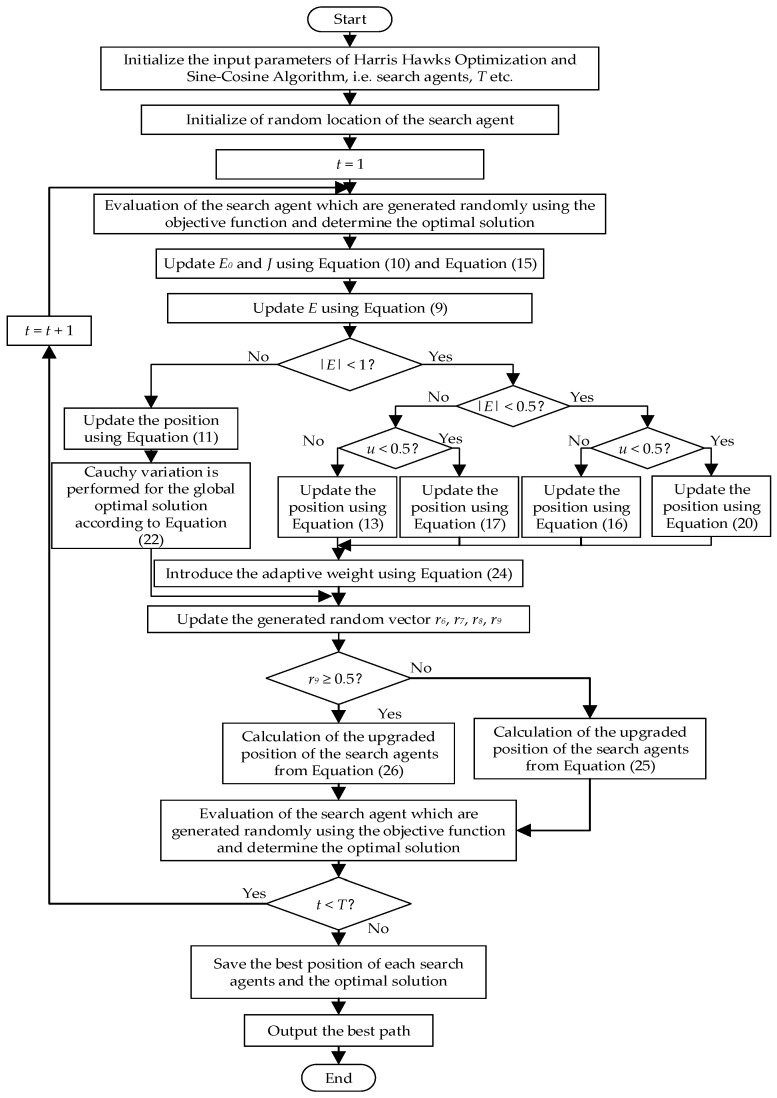
Flowchart of proposed path planning algorithm.

The flowchart of the proposed path planning algorithm based on SCHHO are shown in Figure 2, respectively. 

The implementation steps of the proposed path planning algorithm based on SCHHO are summarized as follows.

**Step 1**: preliminary modeling of a three-dimensional mountain environment.
**Step 2**: initialize the population and parameters *r*_1_, *r*_2_, *r*_3_ and *r*_4_, and calculate the fitness value of each solution.
**Step 3**: calculate the prey energy according to Equation (10). If |*E*| < 1, perform an exploration according to Equation (11) and perform Cauchy variation according to Equation (22) for the global optimal solution produced by Equation (11). If |*E*| ≥ 1, enter local exploitation and judge the besiege mechanisms according to the prey energy *E* and the prey escape probability *u*. In addition, update the prey position and perform local search according to the adaptive weight of Equation (24) and the corresponding besiege formula;
**Step 4**: save the optimal position, perform SCA operation on the position according to Equations (25) and (26), and then change the global optimal position;
**Step 5**: determine whether the number of iterations or iteration precision has been reached. If the number of iterations or iteration precision is not reached, the population and parameters are re-initialized, and the fitness value of each solution is calculated. If it is reached, the optimal path is output.

## 4. Experimental Results and Analysis

### 4.1. Experiment on Benchmark Functions

In order to analyze the performance of SCHHO, benchmark function tests are performed, and several variations of the same classification algorithms are compared. The simulation test environment is: operating system Win11, 64-bit operating system, memory 16 GB, CPU AMD Ryzen 7 5800 H with Radeon Graphics, main frequency 3.20 GHz, simulation software MATLAB 2018b.

#### 4.1.1. Parameter Settings

The modeling mission space has a size of *150 km × 100 km × 20 km* and contains four or five threat regions. The coordinates of start point and end point are set as (10,50,5.57) and (130,10,6.38), respectively. In Section 4.2 below, the improved UAV path planning algorithm and the comparison algorithm are applied to different threat environments. Among them, Case 1–Case 3 proves the superiority of SCHHO algorithm in environments with the same terrain but different threat radius. Case 4 is set up to demonstrate the superiority of the improved algorithm in other different terrain and threat environments. The threat area information of four cases to be tested is presented in Table 1. In order to reflect the fairness and objectivity of the experiment, the population size *N* of all the algorithms is set to 30, the number of iterations *T* is set to 200, and the common parameters of the five algorithms are consistent. The initial parameters of SCHHO are presented in Table 2.

#### 4.1.2. Results and Analysis

Using benchmark functions with different properties is a common approach for the evaluation of the stochastic optimization algorithms. It can ensure that the results obtained by the algorithm are not accidental [36]. Following this fact, several benchmark functions are introduced to verify the validity of the proposed SCHHO method, including unimodal and multimodal benchmark functions [29,37], that are presented in Table 3. In addition, SCHHO is compared with the standard HHO, PSO, SCA and WOA, considering the case of function dimension *n* of 30 and 50. The parameter settings of these algorithms are the same, in order to make a fair comparison in the experimental test.

**Figure 3:**
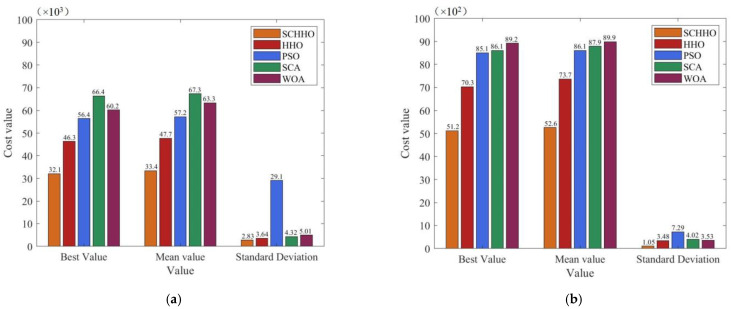

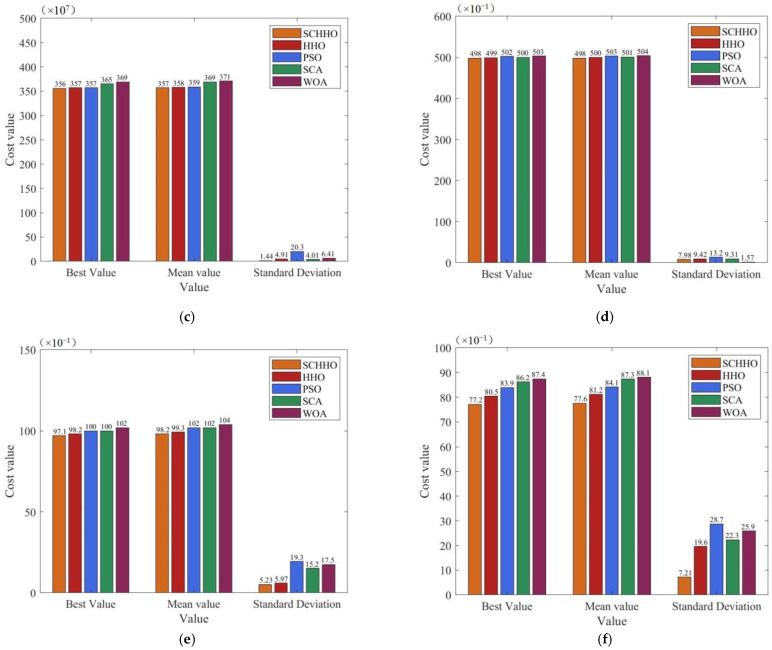
Experimental results on benchmark functions when *n* = 30. (**a**) Sphere. (**b**) Schwefel1.2. (**c**) Rosenbrock. (**d**) Rastrigin. (**e**) Ackley. (**f**) Griewank.

**Figure 4:**
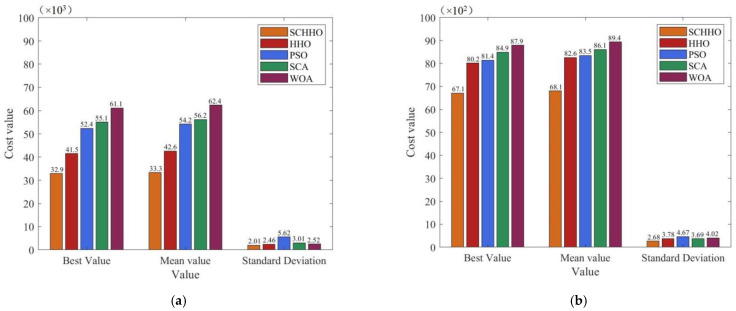

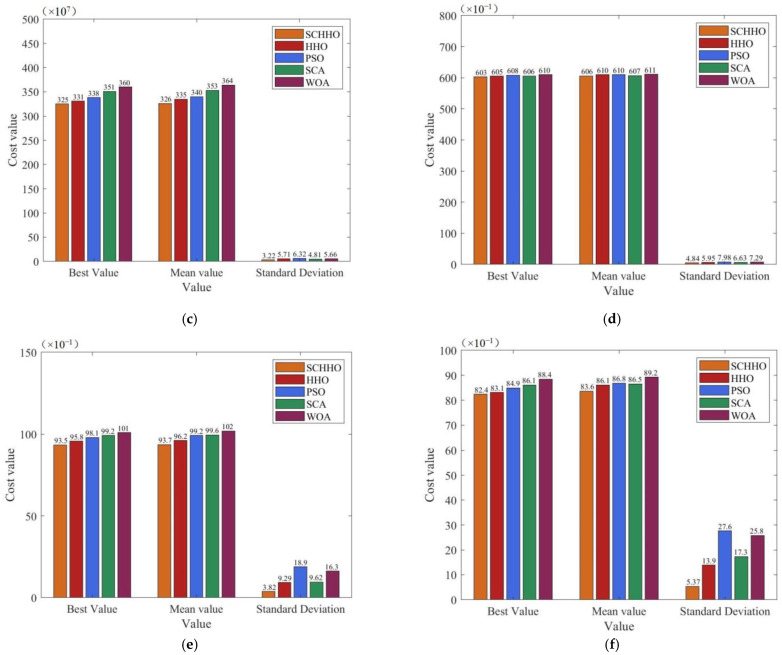
Experimental results on benchmark functions when *n* = 50. (**a**) Sphere. (**b**) Schwefel1.2. (**c**) Rosenbrock. (**d**) Rastrigin. (**e**) Ackley. (**f**) Griewank.

In all the cases, 30-times independent experiments are performed on each benchmark function, and the best value, mean value and standard deviation (Std.) of the objective function values are obtained. With the same benchmark function, the best value, mean value and standard deviation denote the exploration ability, convergence accuracy and stability of the algorithm, respectively. The experimental results are shown in Figure 3 and Figure 4.

It can be seen from Figure 3 and Figure 4 that, after running the five algorithms for 30 times, the best value and mean value obtained by the improved SCHHO are better than those obtained by HHO, PSO, SCA and WOA, for all the six functions. In other words, the convergence accuracy of SCHHO is the highest on the whole. Simultaneously, the standard deviation of the results obtained by SCHHO is smaller than that of the other algorithms, which indicates that SCHHO has a better robustness and the optimal solution is more stable. In summary, the proposed SCHHO improves the exploration and exploitation capacities of the algorithm using the Cauchy mutation strategy and adaptive weight. Thus, it overcomes the problem of low optimization accuracy of the HHO algorithm, greatly improves the optimization performance, and has obvious competitive advantages compared with other existing algorithms.

**Figure A1:**
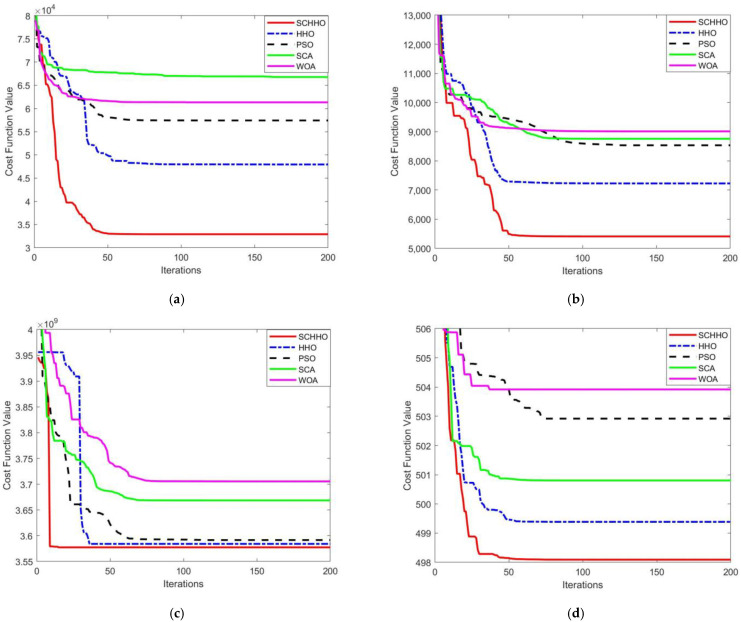

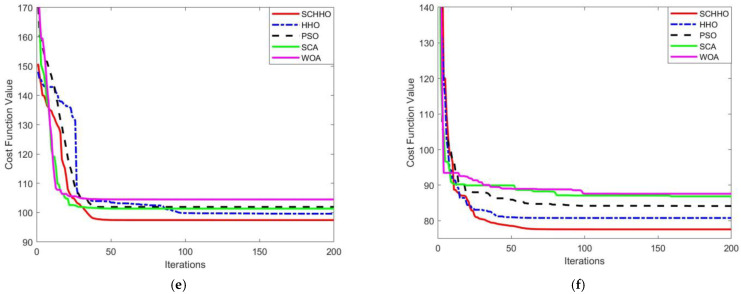
Convergence curves of five algorithms six benchmark functions when *n* = 30. (**a**) Sphere. (**b**) Schwefel1.2. (**c**) Rosenbrock. (**d**) Rastrigin. (**e**) Ackley. (**f**) Griewank.

**Figure A2:**
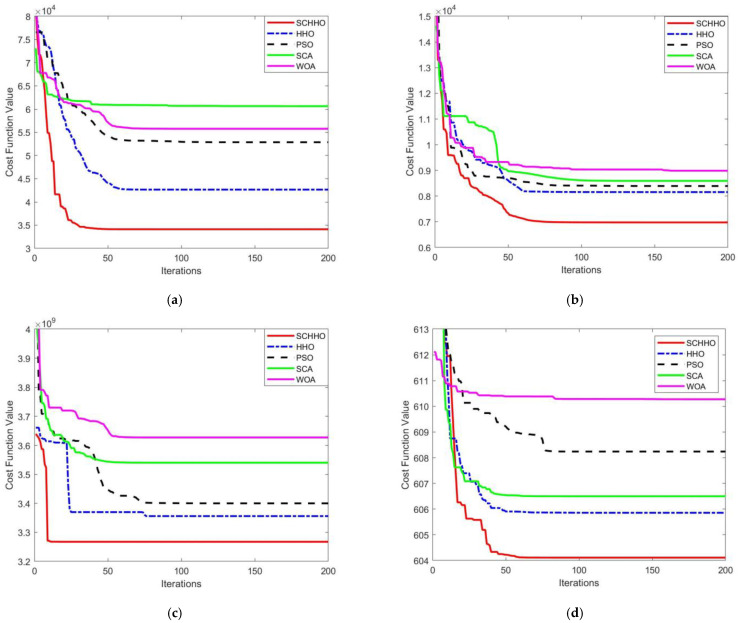

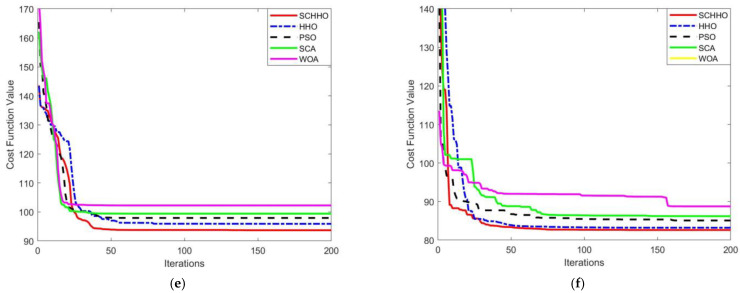
Convergence curves of five algorithms on six benchmark functions when *n* = 50. (**a**) Sphere. (**b**) Schwefel1.2. (**c**) Rosenbrock. (**d**) Rastrigin. (**e**) Ackley. (**f**) Griewank.

In order to intuitively show the optimization performance of SCHHO, the convergence curves of the six benchmark functions for *n* = 30 and *n* = 50 are shown in Figure A1 and Figure A2 in the Appendix A respectively.

It can be seen that the optimization ability of SCHHO is significantly higher than that of PSO, HHO, SCA and WOA, and in the convergence curve of Rosenbroc test function, the convergence speed of SCHHO is obviously higher than that of other algorithms. In the iterative process, multiple inflection points exist in SCHHO. It is proved that due to the introduction of SCA, the improved SCHHO gradually converges to the optimal solution using the oscillation characteristics of the sine and cosine functions, efficiently reduces the possibility of falling into local extreme value, and has a better global optimization effect.

### 4.2. Experiment for Path Planning

Simulation experiments are also performed to evaluate the ability of SCHHO in solving the 3D path planning problem of UAV. Four other algorithms are applied in path planning for comparison: HHO, PSO, SCA and WOA. In all the experiments, the parameters of these algorithms are set according to their original version. For fair comparison, the maximum iteration number of all the algorithms is set to 200, while the population size is set to 30. Considering the randomness of heuristic algorithms, each tested algorithm is independently executed 30 times.

In order to prove the path planning performance in different environments, all the algorithms are applied in four cases. Cases 1–3 are tested for the same threat location but different threat radius. In Cases 1–3, threat regions 1–4 are the active threat areas and threat regions 5–8 are the non-active threat areas. Case 4 is tested for another different terrain and threat environments where threat region 3 and threat regions 5–8 are active threat areas and threat regions 1, 2 and 4 are non-active threat areas. In threat region 3 of Case 4, the radius is 16 km. The threat information of Cases 1–4 has been shown in Table 1.

**Figure 5:**
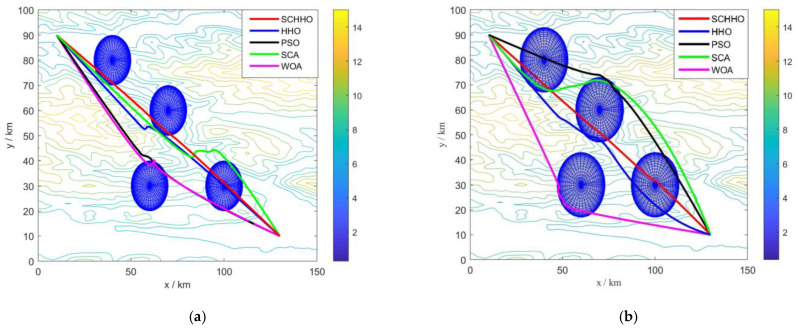

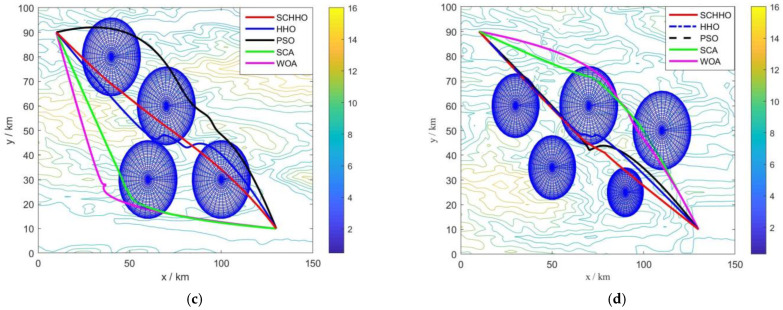
Path planning results in 2D contour map. (**a**) Case 1. (**b**) Case 2. (**c**) Case 3. (**d**) Case 4.

**Figure 6:**
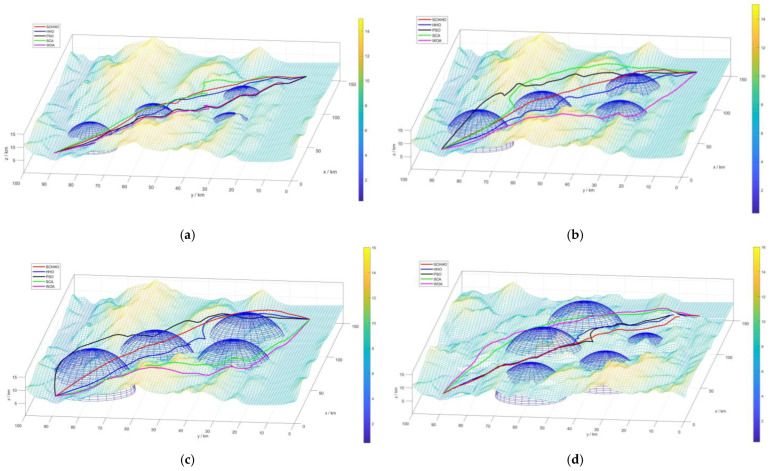
Path planning results in 3D simulation map. (**a**) Case 1. (**b**) Case 2. (**c**) Case 3. (**d**) Case 4.

**Figure 7:**
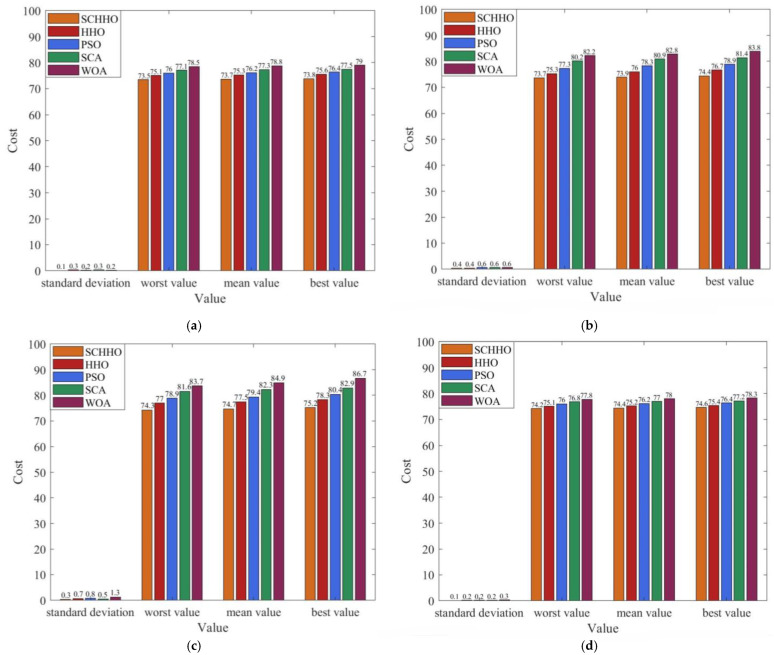
Statistical results of cost function values. (**a**) Case 1. (**b**) Case 2. (**c**) Case 3. (**d**) Case 4.

**Figure 8:**
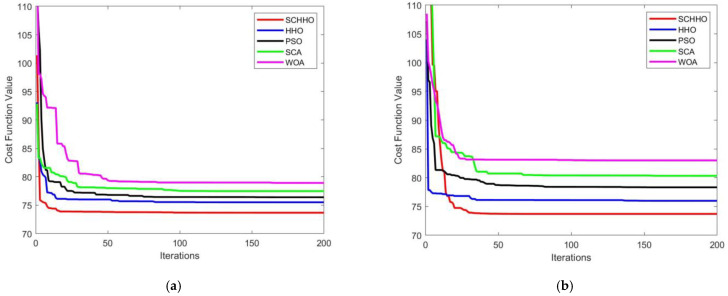

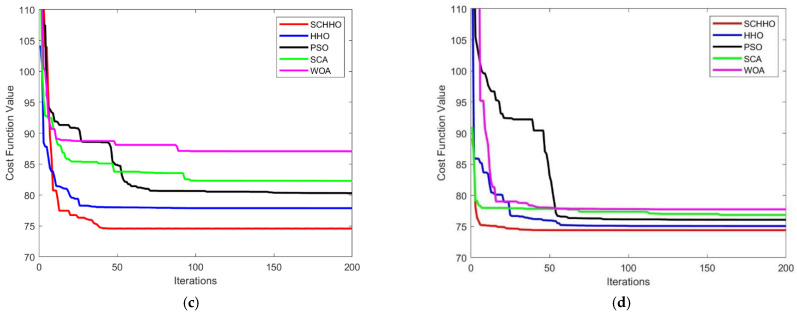
Evolution curves of cost function values. (**a**) Case 1. (**b**) Case 2. (**c**) Case 3. (**d**) Case 4.

Figure 5 presents the 2D contour map, and Figure 6 shows the 3D simulation map of UAV path planning. The UAV starts from the start point and moves forward at a low flying altitude when it is low-lying. When it encounters a steep slope or threat area, it can climb up in accordance with the terrain. After crossing the peak or threat area, it still approaches the target point at a low flying altitude in the valley. The feasibility and efficiency of the proposed algorithm in solving the 3D path planning of UAV are verified. Figure 7 shows the statistical results of cost function values of five algorithms. Figure 8 shows a comparison of the evolution curves of the cost function values for the five algorithms in Case 1 and Case 2, Case 3 and Case 4.

It can be seen from Figure 5 and Figure 6 that among all the five algorithms, SCHHO can find the best path for UAV. It can be seen from Figure 7 that SCHHO has the best performance in terms of the best cost value and mean cost value. In Case 1 to Case 4, the mean value of the improved SCHHO algorithm is 1.06–3.61% higher than that of the basic HHO algorithm, and 3.28–12.01% higher than that of the other three basic algorithms. Meanwhile, the standard deviations of SCHHO algorithm are lower than those of other comparison algorithms. This demonstrates that SCHHO outperforms the other four algorithms in terms of searching ability and stability. It can be seen from Figure 8 that the convergence speed of the SCHHO algorithm performs well in four cases, which is better than most of the other algorithms in this paper. And the greater the threat range, the more prominent the performance of the SCHHO algorithm. In different terrains, the superiority of SCHHO also exists.

**Figure 9:**
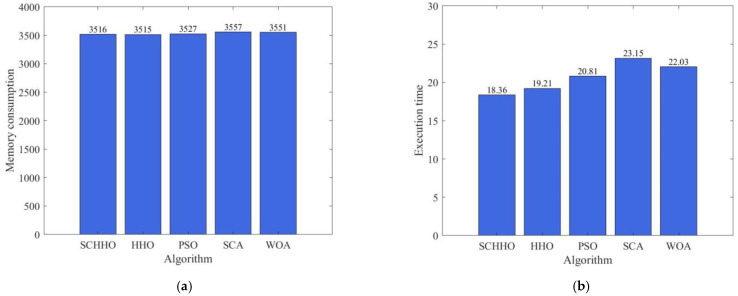
Memory consumption and execution time comparison. (**a**) memory consumption. (**b**) execution time.

In order to compare the execution efficiency of the five algorithms, take Case 1 as a representative to analyze the memory consumption (unit: MB) and execution time (unit: s) of the above five algorithms, as shown in Figure 9.

From Figure 9, it can be seen that the memory consumption of the five algorithms is similar, and SCHHO is slightly better. The SCHHO algorithm runs 4.42–22.2% faster than other algorithms.

**Figure 10:**
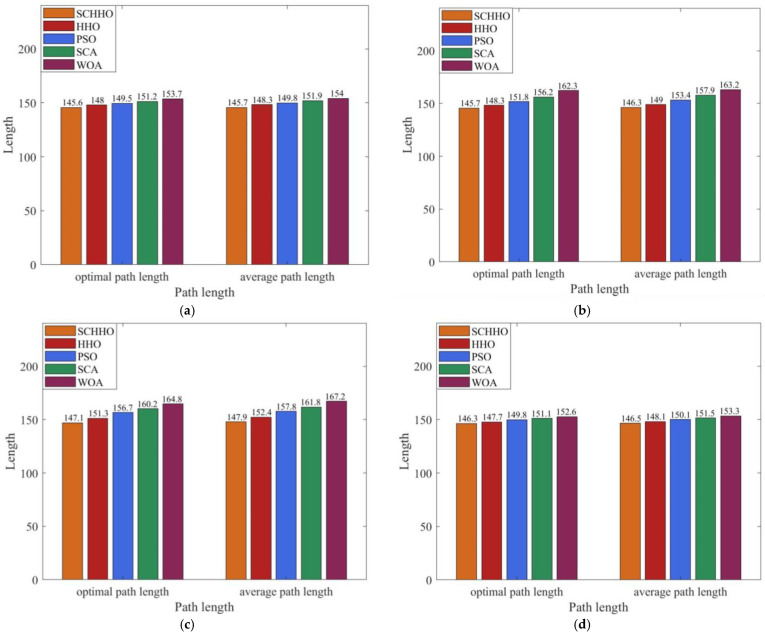
Path length comparison of five algorithms. (**a**) Case 1. (**b**) Case 2. (**c**) Case 3. (**d**) Case 4.

Figure 10 compares the optimal path length and average path length of 100 simulation experiments of the five algorithms in Case 1, Case 2, Case 3 and Case 4. It can be seen that the path planning ability of SCHHO algorithm is better. Among them, the optimal path length of SCHHO is 0.95–10.8% lower than other algorithms, and the average path length is 1.08–11.54% lower than other algorithms. (Unit: km).

The 3D UAV path planning on this complex optimization problem is that the Cauchy distribution function increases the diversity of Harris Hawks population, increases the search space, enhances the global exploration ability of the proposed algorithm, and improves the convergence speed. Simultaneously, an adaptive weight method is introduced to update the neighborhood of prey location and improve the exploitation capacity of the proposed algorithm. In addition, the SCA algorithm improves the convergence ability of the proposed algorithm through its oscillation characteristics. Therefore, the proposed algorithm has good exploration and utilization ability, and it is more competitive in the advantages and stability of optimization objectives, which allows to find the optimal flight path of the UAV.

Generally speaking, maintaining a higher altitude implies an increase in energy consumption. It is assumed that the energy consumption is proportional to the flight altitude, this paper analyzes the energy consumption of five paths under four cases by comparing the Average altitude.

**Figure 11:**
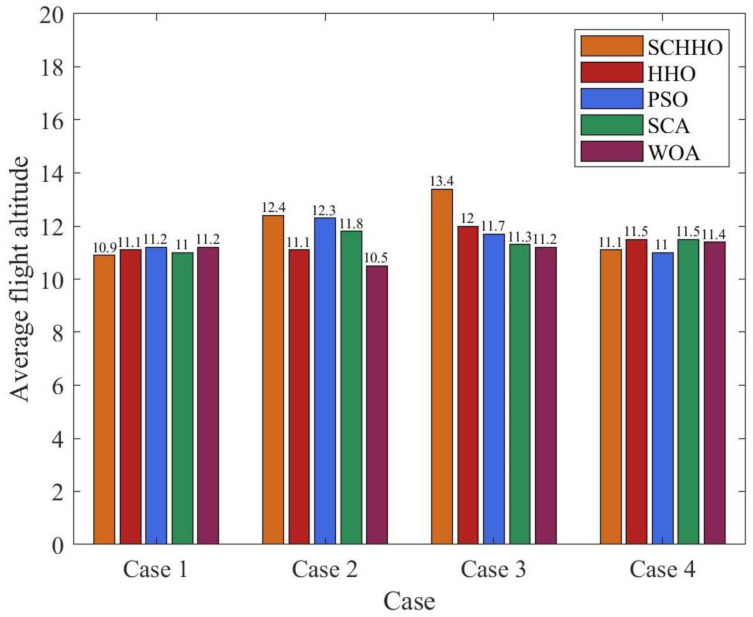
Average height comparison.

It can be seen from Figure 11 that the average height of SCHHO in different environments has little difference with the other four algorithms and in Case 2 and Case 3, the average flight altitude of SCHHO is slightly higher than that of other algorithms. Combined with the comparison of the average path length of the five algorithms in Figure 10, it can be inferred that the energy consumption of the five algorithms is similar.

## 5. Conclusions and Future Work

In this paper, a UAV path planning algorithm based on improved HHO is proposed. A 3D mission space model and flight path cost function are first developed to transform the path planning problem into a multidimensional function optimization problem. HHO is then improved for path planning. In the global exploration stage, the Cauchy distribution function is used to increase the diversity of Harris Hawks population, increase the search space and improve the global exploration ability of the algorithm. In the local exploitation stage, an adaptive weight method is introduced to update the neighborhood of prey location, so as to improve the local exploitation ability. In addition, in order to reduce the possibility of falling into local extremum, SCA is used to gradually converge to the optimal solution. In the experimental results, the standard deviation of the path length planned by SCHHO algorithm is 1.08–11.54% lower than other algorithms, and the average path length is 0.95–10.8% lower than other algorithms, indicating that SCHHO’s path planning ability and stability are higher than other algorithms. In addition, the improved SCHHO algorithm runs 4.42–20.7% faster than other algorithms. The obtained results demonstrate that the proposed algorithm has certain advantages in dealing with path planning problems in 3D mountain environments with multiple threat ranges. Many challenges remain in future work, such as dynamic barriers and unknown interference. Thus, multi-UAV mission assignment and path planning issues with dynamic threats will continue to be focus on. Moreover, it would be meaningful to constantly improve the equipment and facilities, and strive to apply the research results in the actual environment. 

## Figures and Tables

**Table 1:** The threat information of Case 1–4.

Name	Coordinates	Radius
threat region 1	(40,80,0)	10/13/16
threat region 2	(60,30,0)	10/13/16
threat region 3	(70,60,0)	10/13/16
threat region 4	(100,30,0)	10/13/16
threat region 5	(30,60,0)	13
threat region 6	(50,35,0)	13
threat region 7	(90,25,0)	10
threat region 8	(110,50,0)	16

**Table 2:** Initial parameter of SCHHO.

Parameter	Meaning	Value
*ω*_1_	Weight coefficient of path length	0.5
*ω*_2_	Weight coefficient of average flight height	0.3
*ω*_3_	Weight coefficient of comprehensive threat index	0.2
*T*	Maximum iteration	200
*N*	Population size	30
*D*	Problem dimension	30
*l*_min_	Minimum path	130
*L*_max_	Maximum path	200
*h*_min_	Minimum Ground clearance	5
$\Delta \varphi_{\max}$	Maximum turning angle	270
$\theta_{\max}$	Maximum climb angle	90

**Table 3:** Six benchmark functions.

Name	Definition	Domain	Minimum
Sphere	$f_{1}(x)=\sum_{i=1}^{D}x_{i}^{2}$	[−100,100]	0
Schwefel 1.2	$f_{2}(x)={\sum_{i=1}^{n}\left(\sum_{j-1}^{i}x_{j}\right)}^{2}$	[−100,100]	0
Rosenbrock	$f_{3}(x)=\sum_{i=1}^{n-1}\left[100{\left(x_{i+1}-x_{i}^{2}\right)}^{2}+{\left(x_{i}-1\right)}^{2}\right]$	[−100,100]	0
Rastrigin	$f_{4}(x)=\sum_{i=1}^{D}\left[x_{i}^{2}-10\cos(2\pi x_{i})+10\right]$	[−5.12,5.12]	0
Ackley	$f_{5}(x)=20-20\exp\left(-\frac{1}{5}\sqrt{\frac{1}{n}\sum_{i=1}^{n}x_{i}^{2}}\right)-\exp\left(\frac{1}{n}\sum_{i=1}^{n}\cos\left(2\pi x_{i}\right)\right)+e$	[−32,32]	0
Griewank	$f_{6}(x)=\sum_{i=1}^{n}\frac{x_{i}^{2}}{4000}-\prod_{i=1}^{n}\cos(\frac{x_{i}}{\sqrt{i}})+1$	[−100,100]	0

## Data Availability

Not applicable.
